# CRISPR-Cas12a combination to alleviate the false-positive in loop-mediated isothermal amplification-based diagnosis of *Neisseria meningitidis*

**DOI:** 10.1186/s12879-022-07363-w

**Published:** 2022-05-04

**Authors:** Ngo Tat Trung, Le Huu Phuc Son, Trinh Xuan Hien, Dao Thanh Quyen, Mai Hong Bang, Le Huu Song

**Affiliations:** 1 Centre for Genetics Consultation and Cancer Screening (CGC), Hanoi, Vietnam; 2 Vietnamese-German Center for Medical Research (VG-CARE), Hanoi, Vietnam; 3 Faculty of Tropical and Infectious Diseases, 108 Military Central Hospital, Hai Ba Trung District, No 1, Tran Hung Dao Street, Hanoi, Vietnam; 4 108 Institute of Clinical Medical and Pharmaceutical Sciences, Hanoi, Vietnam

**Keywords:** *Nesseria menitigistis*, LAMP, PCR, CRISPR, Cas12a

## Abstract

**Background:**

Loop isothermal amplification (LAMP) has recently been proposed as a point-of-care diagnostic tool to detect acute infectious pathogens; however, this technique embeds risk of generating false-positive results. Whereas, with abilities to accurately recognize specific sequence, the CRISPR/Cas12a can forms complexes with cognate RNA sensors and cleave pathogen’s DNA targets complimerntary to its cognate RNA, afterward acquiring the collateral activity to unbiasedly cut nearby off-target fragments. Therefore, if relevant fluorescent-quencher-nucleic probes are present in the reaction, the non-specific cleavage of probes releases fluorescences and establish diagnostic read-outs.

**Methods:**

The MetA gene of *N. meningitidis* was selected as target to optimize the LAMP reaction, whereas pseudo-dilution series of *N. meningitidis* gemonics DNA was used to establish the detection limit of LAMP/Cas12a combination assay. The diagnostic performance of established LAMP/Cas12a combination assay was validated in comparation with standard real-time PCR on 51 CSF samples (14 N*. meningitidis* confirmed patients and 37 control subjects).

**Results:**

In relevant biochemical conditions, CRISPR-Cas12a and LAMP can work synchronously to accurately identify genetics materials of *Nesseria menitigistis* at the level 40 copies/reaction less than 2 h.

**Conclusions:**

In properly optimized conditions, the CRISPR-Cas12a system helps to alleviate false positive result hence enhancing the specificity of the LAMP assays.

**Supplementary Information:**

The online version contains supplementary material available at 10.1186/s12879-022-07363-w.

## Background

*Neisseria meningitidis* is a Gram-negative bacterium that causes severe meningitis and sepsis. These diseases require fast and accurate diagnostics to indicate proper antimicrobial therapies [1, 2]. So far, together with blood culture, polymerase chain reaction (PCR) is recommended as a routine technique for the diagnostic confirmation [3, 4]. PCR requires laboratories with sophisticated infrastructures and well-trained personnel, therefore making challenges for deploying in limited-resource areas. Loop-mediated isothermal amplification (LAMP) based approaches have been used to detect pathogens [5]. LAMP-based assays are faster and require no sophisticated instruments or/and skilled personnel, therefore having the advantage to use as on-site diagnostic device [6].

LAMP can achieve PCR’s sensitivity without complicated thermocycling, some LAMP assays can be completed within 30 min. However, LAMP detection step acquires non-specific indicators (such as Mg^2+^, intercalating dyes, labelled primers) that cannot distinguish spurious amplicons [7–9]. We documented several phenomena that real-time PCR protocols [4, 10] could not recapitulate positive results gained by LAMP reactions and some LAMP positive cases lacked meningitidis specific clinical symptoms [1].

We suspected that LAMP assay might embed risks of generating false positive [7–9]. Whereas, with abilities to accurately recognize specific sequences, the CRISPR-Cas system holds promising potentials to tackle the above-mentioned problem: In this system, the DNAse cas12a forms a complex with their cognate CRISPR RNAs to induce the cleavage of pathogen’s RNA or DNA in a sequence-specific manner. Afterwards, the collateral transcleavage activity is also triggered to unbiasedly cut the nearby off-target fragments. If relevant fluorescent-quencher-nucleic probes are present in the reaction, the non-target cleavage of probes will release fluorescent signals and establish diagnostics read-outs [11–13]. Thus, in this study, we have established an effective method combining the LAMP assay and the CRISPR-Cas12a system for the diagnosis of patients infected with *Neisseria meningitidis.*

## Results

**Fig. 1:**
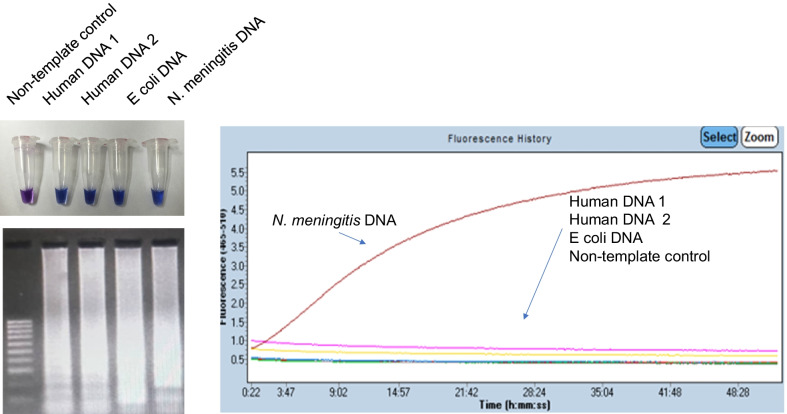
Addition of CRISPR-Cas12a help to alleviate false positive acquired by single use of LAMP performance: (upper left panel) the product mixture of LAMP assay was colometric indicated by addition of 100 uM Hydroxy naphthol blue (HNB Sigma—Singapore) or (downer left panel) resolved against 1.2% agarose gel electrophoresis. Right panel—the same product mixture of LAMP was treated with CRISPR-Cas12a

We first performed isothermal amplification assay using Bst DNA Polymerase with primers specific for *MetA* gene of *N. meningitidis*. The reaction products were resolved against 1.5% agarose gel. It is impossible to distinguish the electrophoretic banding pattern between human DNA, *E. coli* DNA or *N. meningitidis* DNA (Fig. 1 upper-left panel and Additional file 1: Fig. S1). However, once these products were treated with CRISPR-Cas12a with gRNA sequence complementary to *MetA* gene of *N. meningitidis*, only fluorescent signals from samples with *N. meningitidis* DNA was recorded. Thus, treatment of CRISPR-Cas12a helps to alleviate false-positive results by single-use of LAMP assay.

**Fig. 2:**
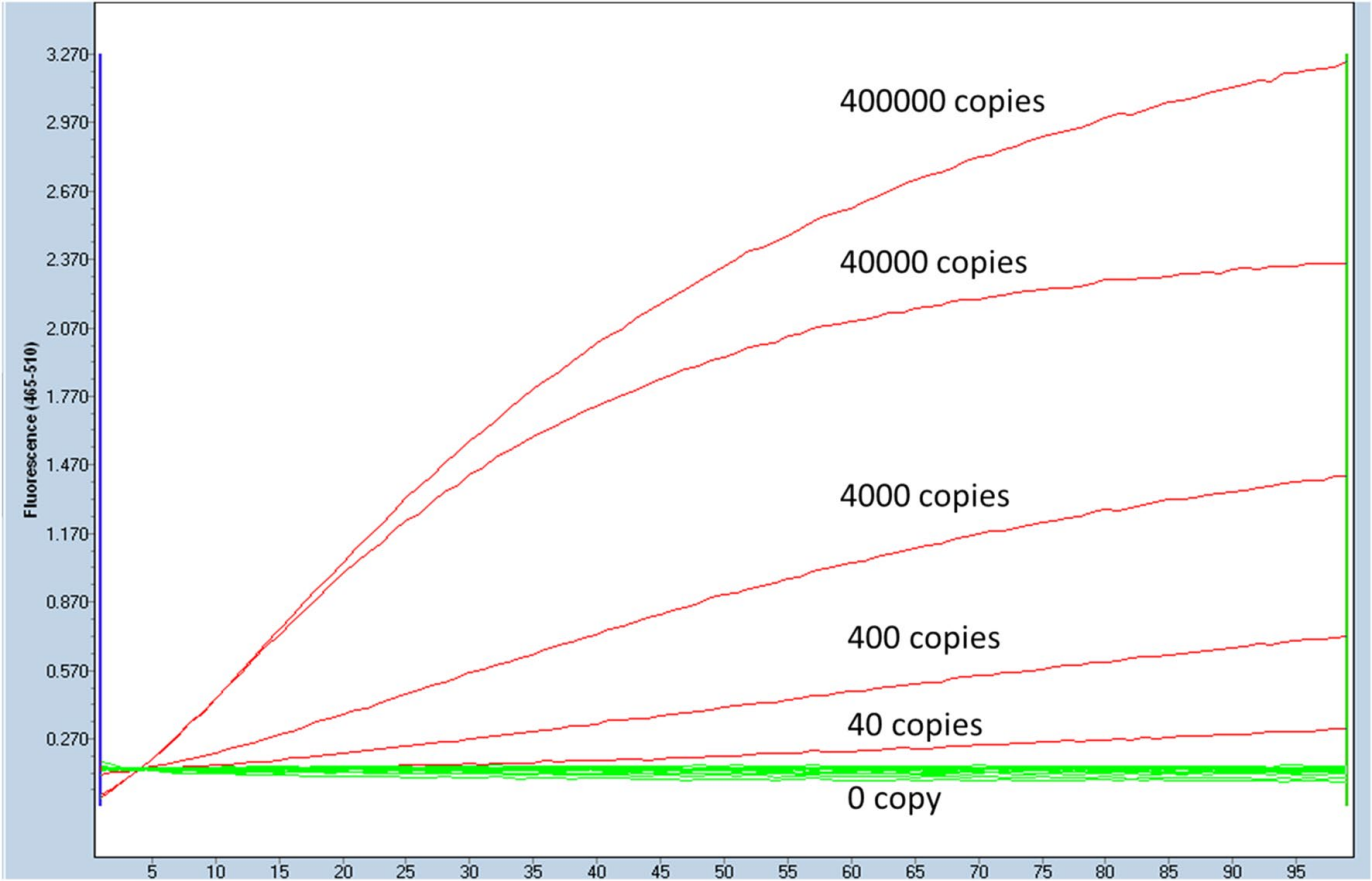
Detection limit and diagnostics performance of LAMP/CRISPR-Cas12a for identification of *N. meningitidis* DNA. Left panel: Detection limit of LAMP/CRISPR-Cas12a for identification of *N. meningitidis* DNA: the fluorescent signals acquired by Bst DNA Polymerase based isothermally amplifying at 55 °C for 30 min on pseudo-samples of 0, *40, 400, 4000, 40,000 and 400,000 and 400,000 copies of N. menitigitidis* PCR amplicon spiked into 25 mM Tris–EDTA pH 8 containing the background of 10^8^ copies *E. coli* PCR amplicon

To evaluate the detection limit of LAMP/CRISPR-Cas12a combination for the detection of *N. meningitidis* DNA, we spiked series of 0, 40, 400, 4,000, 40,000 and 400,000 copies of *N. meningitidis* PCR amplicon into 25 mM Tris–EDTA pH 8 containing the background of 10^8^ copies of *E. coli* PCR amplicon/ul (Additional file 2). These dilution points were used as the templates for Bst DNA Polymerase isothermal amplification at 55 °C for 45 min then treated with CRISPR-Cas12a (1.0 µM Cas12a per reaction), in the presence of 0.25 µM guide RNA and 0.25 µM fluorescence labelled reporter at 55 °C for 30 min; the fluorescent signal was recorded in 510 nm in Roche light cycler 480. The fluorescence was detected at all prepared dilution points even at the lowest level of 40 copies of *N. meningitidis* (Fig. 2).

To validate the clinical performance, we applied the newly established procedure to identify *N. meningitidis* from 51 CFS samples from *N. meningitidis* suspected patients. The standard conventional realtime PCR assay with *MetA* genes as a molecular target was also used to confirm the presence of *N. meningitidis* DNA. Realtime PCR identified *N. meningitidis* DNA from 13 out of 51 recruited CFS samples (Additional file 1: Fig. S3). Whereas, single-use of LAMP assay identified 18 cases positive with *N. menitigitidis*, in which, five cases did not match to either result acquired by real-time PCR or patients’ clinical symptoms and were considered as false positive. However, when LAMP reaction mixtures from 51 CFS samples were treated with CRISPR-Cas12a, only 13 cases were positive. Importantly, all of these 13 cases were matched to the results gained by conventional PCR (Table 1).

**Table 1:** *O*ligonucleotides used as primers to loop-mediated-isothermally amplify the MetA target of *N. meningitidis*

Oligo names/concentration	Sequences (5′–3′)	Volume uses for one reaction
Tr-Hien-metA-F3(10 pmol/μl)	GCAGTTCCTAATTTACCATGA	0.5 µl 0.5 µl
Tr-Hien-metA-B3(10 pmol/μl)	GCAACGAAAATTGCAACTGTA	
Tr-Hien-metA-FIP(40 pmol/μl)	GGTGAATTTGTTCCCATTATTGCGCACCATGATACCCCCATG	0.75 µl 0.75 µl
Tr-Hien-metA-BIP(40 pmol/μl)	TTCACATTTTGGCTGTCAAAGGCTATGATGATTACACCTGT	
Tr-Hien-metA-LF(10 pmol/μl)	GCTGCTTTTGGCGGTGCATT	1 µl 1 µl
Tr-Hien-metA-LB(10 pmol/μl)	CTTGGCTGTCTAAATTTTGCGC	
TR-H-VapA-NM-gRNA	UAAUUUCUACUAAGUGUAGAUAGCCUGUGAUAAUUGAAUUGC	

## Discussion

Single-use of LAMP embeds high risk of false-positive signals that challenge the employing LAMP-based assays into clinical practices [7, 9]. However, LAMP posscess strong intrinsic amplification potential and it is simple to operate, hence would benefit the communities if the LAMP’s weaknesses are somehow solved. Our data revealed that the sequential treatment LAMP products by CRISPR-Cas12a under the guidance of specific gRNA sequence can abolish non-specific signals. This technical integration of two enzymes, in one side help to sustain the strong amplification potential of LAMP on the other side, significantly enhances the specificity of diagnostic procedures.

Previous study has reported that trehalose is an exceptional protein thermal stability stabilizer [14], and we also believe and can recapitulate the previous findings (Additional file 1: Fig. S2) and found that trehalose helps CRISPR-Cas12a to sustain their activity in a single trehalose containing buffer at 55 °C (Additional file 1: Fig. S2). This condition omits buffer replacement from the LAMP into CRISPR assay thereby reducing sample handling and contamination risk. However, in various tested biochemical environments, CRISPR-Cas12a strongly inhibits isothermal DNA polymerases (Bst and Bsu), therefore, we were not successful in coupling isothermal enzymes and CRISPR-Cas12a into single reaction tubes. Further studies are needed to mitigate the inhibitory effect of CRISPR-Cas12a to Bst or Bsu DNA polymerase, thereby combining CRISPR-Cas12a with isothermal amplification into single tube diagnostic device.

## Conclusion

The sequential combination of LAMP and CRISPR-Cas12a can alleviate false-positive acquired by single use of LAMP performance.

## Supplementary Information

**Additional file 1.** Study design and supplementary data.

**Additional file 2.** Experiment procedure.

## Data Availability

Data and supporting materials associated with this study will be shared upon request.

## Abbreviations

LAMP: Loop isothermal amplification
CRISPR: Clustered regularly interspaced short palindromic repeats
PCR: Polychain reaction
