# Supplementary material for: CRISPR-Cas12a combination to alleviate the false-positive in loop-mediated isothermal amplification-based diagnosis of Neisseria meningitidis
Source: BMC Infect Dis. 2022 May 4;22:429. doi: 10.1186/s12879-022-07363-w (PMC9066958; doi:10.1186/s12879-022-07363-w)
Supplement: Supplementary file 1 — Additional file 1. Study design and supplementary data. [file 12879_2022_7363_MOESM1_ESM.pptx]

## Slide 1
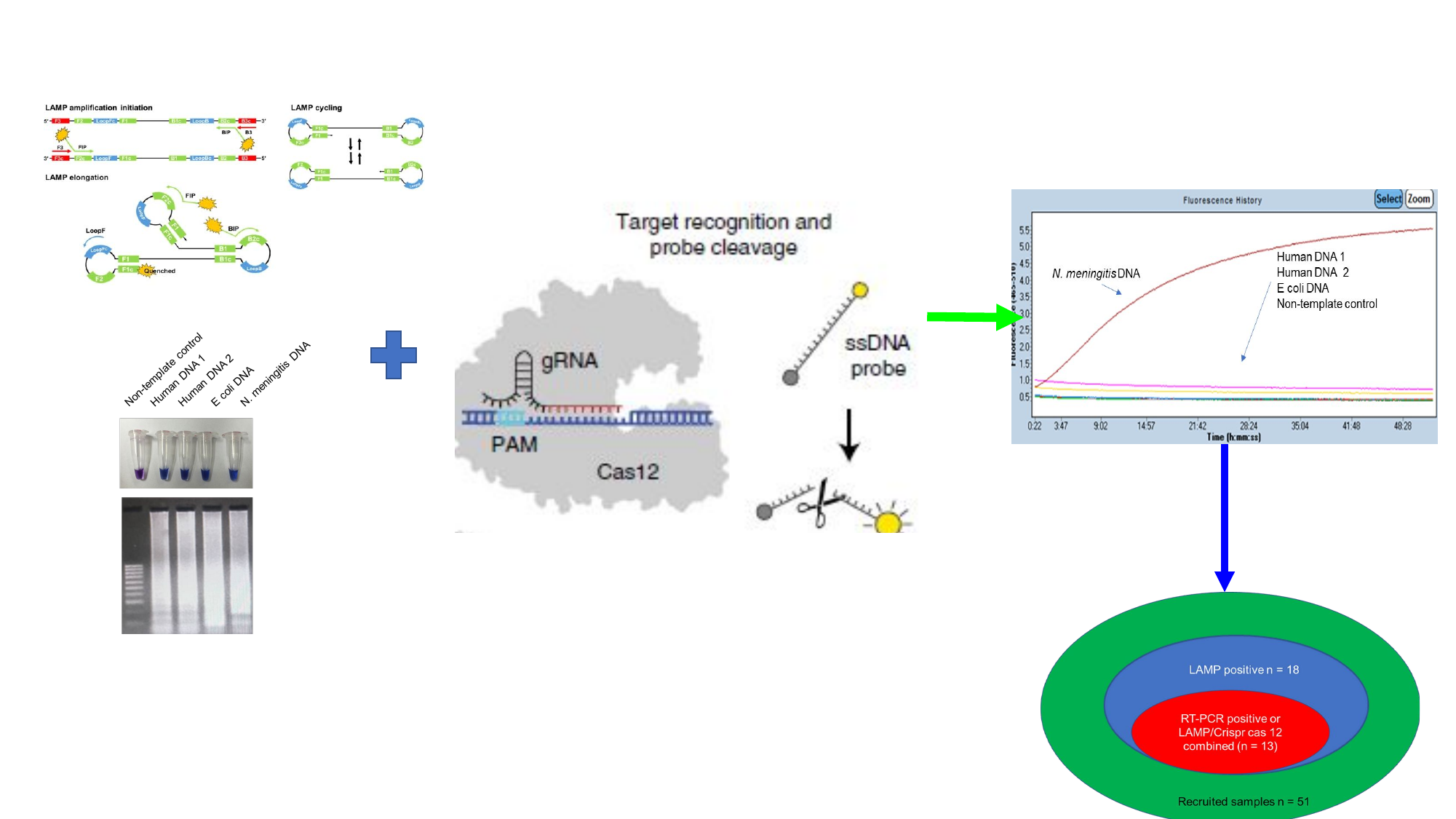

## Slide 2
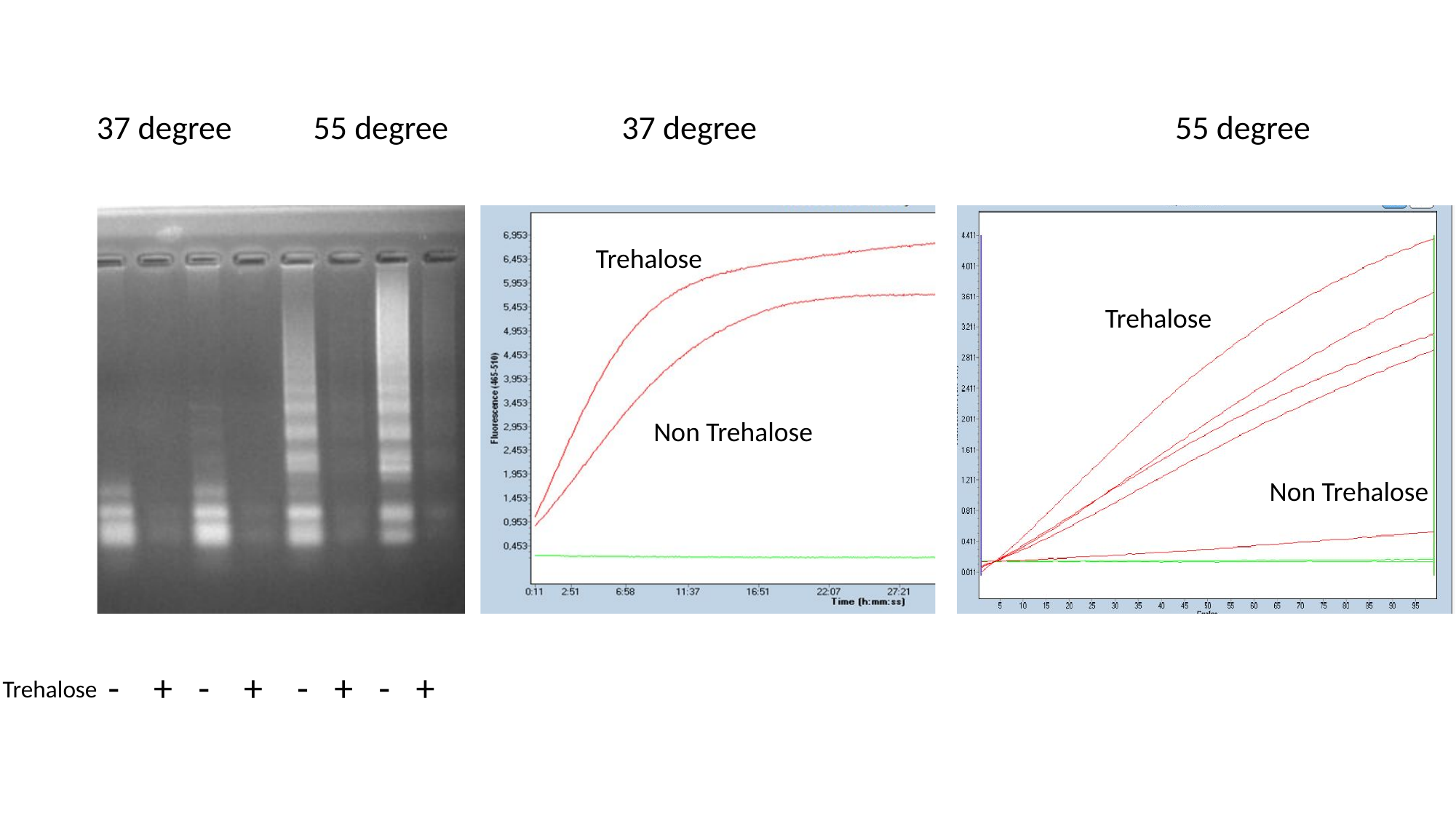

37 degree
55 degree
37 degree
55 degree
Trehalose
Trehalose
Non Trehalose
Non Trehalose
- + - + - + - +
Trehalose

## Slide 3
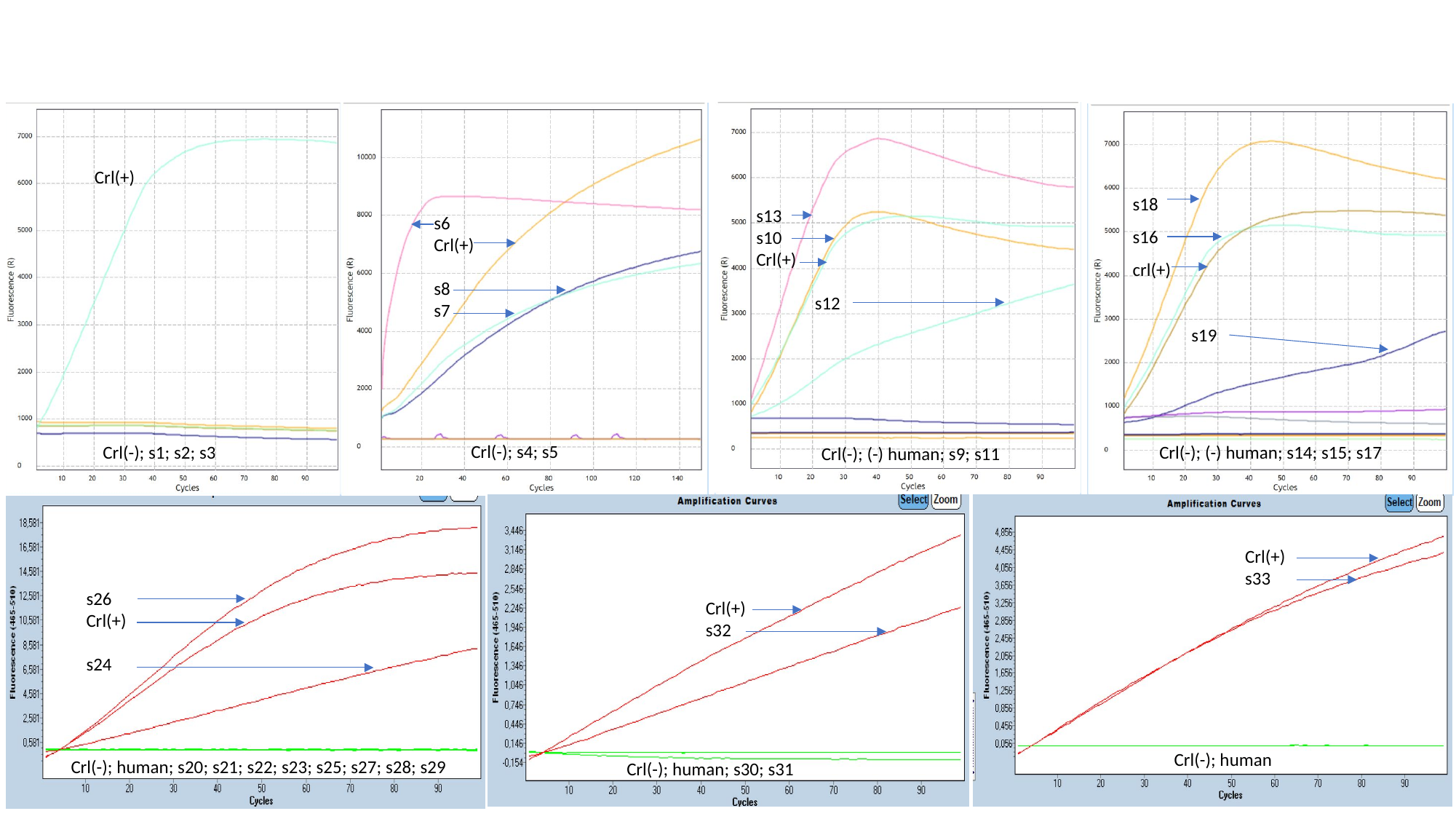

Crl(+)
s18
s16
crl(+)
 s19
s13
s10
Crl(+)
 s12
s6
Crl(+)
s8
s7
Crl(-); s4; s5
Crl(-); s1; s2; s3
Crl(-); (-) human; s14; s15; s17
Crl(-); (-) human; s9; s11
Crl(+)
s33
s26
Crl(+)
s24
Crl(+)
s32
Crl(-); human
Crl(-); human; s20; s21; s22; s23; s25; s27; s28; s29
Crl(-); human; s30; s31
