# Supplementary material for: CRISPR-Cas12a combination to alleviate the false-positive in loop-mediated isothermal amplification-based diagnosis of Neisseria meningitidis
Source: BMC Infect Dis. 2022 May 4;22:429. doi: 10.1186/s12879-022-07363-w (PMC9066958; doi:10.1186/s12879-022-07363-w)
Supplement: Supplementary file 2 — Additional file 2. Experiment procedure. [file 12879_2022_7363_MOESM2_ESM.docx]

Short Communication

**CRISPR-Cas12a combination to alleviate the false-positive in loop-mediated isothermal amplification-based diagnosis of *Neisseria meningitidis***

Ngo Tat Trung^1,2,3,4#^, Le Huu Phuc Son^1,2*^, Trinh Xuan Hien^1,2*^, Dao Thanh Quyen^2,3,4^, Mai Hong Bang^2,4^ and Le Huu Song^2,3,4#^

**Corresponding author: Dr. Ngo Tat Trung, PhD**

Faculty of Tropical and Infectious Diseases

Centre for Genetics Consultation and Cancer Screening (CGC)

Vietnamese-German Center for Medical Research (VG-CARE),

108 Institute of Clinical Medical and Pharmaceutical Sciences

No 1, Tran Hung Dao Street,

Hai Ba Trung District, Hanoi, Vietnam

Tel. +84 919119416

Fax. +84 439728027

Email: [tatrungngo@gmail.com](mailto:tatrungngo@gmail.com)

**Co-first author:**

**Le Huu Phuc Son**

Faculty of Tropical and Infectious Diseases

Vietnamese-German Center for Medical Research (VG-CARE),

108 Institute of Clinical Medical and Pharmaceutical Sciences No 1, Tran Hung Dao Street

Hai Ba Trung District, Hanoi, Vietnam

**Co-first author:**

**Trinh Xuan Hien**

Faculty of Tropical and Infectious Diseases

Vietnamese-German Center for Medical Research (VG-CARE),

108 Institute of Clinical Medical and Pharmaceutical Sciences

No 1, Tran Hung Dao Street,

Hai Ba Trung District, Hanoi, Vietnam

**Contribution author:**

**Dao Thanh Quyen**

Faculty of Tropical and Infectious Diseases

Vietnamese-German Center for Medical Research (VG-CARE),

Department of Molecular Biology

108 Institute of Clinical Medical and Pharmaceutical Sciences No 1, Tran Hung Dao Street

Hai Ba Trung District, Hanoi, Vietnam,

**Mai Hong Bang**

Faculty of Gastroenterology

Vietnamese-German Center for Medical Research (VG-CARE),

108 Institute of Clinical Medical and Pharmaceutical Sciences

No 1, Tran Hung Dao Street,

Hai Ba Trung District, Hanoi, Vietnam

**Email**: [bangmh@benhvien108.vn](mailto:bangmh@benhvien108.vn)

**Co-Corresponding author: Assoc. Prof. Dr. Le Huu Song, MD, PhD**

Faculty of Tropical and Infectious Diseases

Vietnamese-German Center for Medical Research (VG-CARE),

108 Institute of Clinical Medical and Pharmaceutical Sciences

No 1, Tran Hung Dao Street,

Hai Ba Trung District, Hanoi, Vietnam

Tel. +84 69 698713

Fax. +84 439728027

**Email.** [lehuusong@108-icid.com](mailto:lehuusong@108-icid.com)

^1^ Centre for Genetics Consultation and Cancer Screening (CGC)

^2^ Vietnamese-German Center for Medical Research (VG-CARE), Hanoi, Vietnam

^3^ Faculty of Tropical and Infectious Diseases, 108 Military Central Hospital, Hanoi, Vietnam

^4^108 Institute of Clinical Medical and Pharmaceutical Sciences

^(#)^ These authors equally contribute to the work

^(*)^ These authors equally contribute to the work

# Materials and methods

**Ethic approval:** The study was submitted for regulatory approval to the Institutional Review Board of the 108 Military Central Hospital in Hanoi and was approved. The Ethical Committee of the 108 Military Central Hospital, Hanoi, provided ethical approval for the study. Informed written consent was obtained from all study participants or from their parents/guardians if the study participant was in an unconscious condition.

**Sample processing and DNA extraction:** A total of 0.3 ml CSF samples from 14 *N. meningitidis* confirmed patients and 37 control subjects were mixed with 300 μl universal lysis solution (50 mM Tris-HCl pH 7.5, 0.5% Triton-X100) and heated for 5 minutes at 65°C and centrifuged at 13,000 g for five minutes. The upper aqueous supernatant was collected into an Eppendorf tube and 5ul of collected supernatant was used as template for Loop-mediated-isothermal amplification described before[1].

## Loop-mediated-isothermal amplification (LAMP) was designed as described previously [1] in which oligonucleotides below (table 1) were used to isothermally amplify the MetA target of N. meningitidis at 55^o^C for 45 minutes in buffer containing (20 mM TrisHCl pH 8.3, 10 mM CH_3_COONH_4_, 2 mM (CH_3_COO)_2_Mg_2_, 1% Tween20, 0.5 mg/ml Casein, 320 mM Trehalose) with the catalysis by 8 units of Bst DNA Polymerase (New England Biolabs) per reaction. The product mixture of LAMP assays was colormetric indicated by addition of 100 uM Hydroxy naphthol blue (HNB Sigma - Singapore) or resolved against 1.2% agarose gel electrophoresis.

***CRISPR-Cas12 assay:*** 3 ul of the LAMP product was mixed with 2 ul buffer containing (20mM TrisHCl pH 8.3, 10mM CH_3_COONH_4_, 2 mM (CH_3_COO)_2_Mg_2_, 1% Tween20, 0.5 mg/ml Casein, 320mM Trehalose) and 1.0 µM EnGen® Lba Cas12a (Cpf1 - New England Biolabs), 0,25 µM guideRNA (UAA UUU CUA CUA AGU GUA GAU AGC CUG UGA UAA UUG AAU UGC) and 0,25 µM fluorescence labelled reporter (FAM-TTATT-IABkFQ). The reaction mixture was incubated at 55^o^C for 30 minutes and fluorescent signal was recorded in 510 nm in Roche light cycler 480.

## Real-time PCR conditions: The real-time PCR assay mixtures consisted of 7.5 µl Taqman real-time PCR master mix (Qiagen, Hilden, Germany), 5 µl of DNA template, 5 pmol of primers TR-H-VapA-NM-F: GCA GTT CCT AAT TTA CCA TG/ TR-H-VapA-NM-R: GCG AAT TTG CTA ATC CTA TTT ATG TGC and 0.2 pmol of probes TR-H-VapA-NM-Probe: FAM-AAC CAG CGC AAC GAA AAT TGC AA. Reactions were run in the Roche light cycler 480 device with a pre-incubation step at 50^°^C for 15 min, initial denaturation at 95^°^C for 5 min, followed by 45 cycles of 95^°^C for 15 sec and 60^°^C for 60 sec.

**Serial dilution formulation:** To make pseudo-dilution series of *N. meningitidis* gemonics DNA against the control pathogens, we first generated 500 bp PCR amplicon amplified from *N. meningitidis* genomics DNA (we called *N. meningitidis* PCR amplicon) using primer pairs **tgc att aat tat tag cag cat tgg cac caa/ aat tat tga ctg aca agg gta aat tca aac** targeting *MetA* gene and PCR amplicon amplified from *E. coli* genomics DNA using primer pairs acc tct ctt acc tgc gct gca tac cgg aaa/ggc gcg cat ttc ttc ctg agt tcc ttg tgg that targets Dx gene (we called *E. coli* PCR amplicon). Afterward, *N. meningitidis* PCR amplicon and *E. coli* PCR amplicon were purified separately (using PCR purification kit from Thermofisher) equalized in molar. From these, the pseudo-dilution series of *N. meningitidis* against *E. coli* was made by formulating series of 0, 40, 400, 4000, 40000 and 400000 copies of *N. meningitidis* PCR amplicon into 25 mM Tris-EDTA at pH 8 containing the background of 10^8^ copies *E. coli* PCR amplicon/ul. These dilution points were later used to validate the detection limits of LAMP and LAMP/CRISPR combined assays.

# References

1. Notomi T, Okayama H, Masubuchi H, Yonekawa T, Watanabe K, et al. (2000) Loop-mediated isothermal amplification of DNA. Nucleic Acids Res 28: E63.

## Table 1: Oligonucleotides used as primers to loop-mediated-isothermally amplify the MetA target of N. meningitidis

| Oligo names/concentration | Sequences (5’-3’) | Volume uses for one reaction |
| --- | --- | --- |
| Tr-Hien-metA-F3(10pmol/ul) | GCAGTTCCTAATTTACCATGA | 0.5 µl  0.5 µl |
| Tr-Hien-metA-B3(10pmol/ul) | GCAACGAAAATTGCAACTGTA |  |
| Tr-Hien-metA-FIP(40pmol/ul) | GGTGAATTTGTTCCCATTATTGCGCACCATGATACCCCCATG | 0.75 µl  0.75 µl |
| Tr-Hien-metA-BIP(40pmol/ul) | TTCACATTTTGGCTGTCAAAGGCTATGATGATTACACCTGT |  |
| Tr-Hien-metA-LF(10pmol/ul) | GCTGCTTTTGGCGGTGCATT | 1 µl  1 µl |
| Tr-Hien-metA-LB(10pmol/ul) | CTTGGCTGTCTAAATTTTGCGC |  |
|  |  |  |
